# Commentary: A Human Pluripotent Stem Cell-Based Platform to Study SARS-CoV-2 Tropism and Model Virus Infection in Human Cells and Organoids

**DOI:** 10.3389/fendo.2020.585922

**Published:** 2020-10-14

**Authors:** Amin Ardestani, Kathrin Maedler

**Affiliations:** ^1^ Centre for Biomolecular Interactions Bremen, University of Bremen, Bremen, Germany; ^2^ Department of Molecular Medicine, School of Advanced Technologies in Medicine, Tehran University of Medical Sciences, Tehran, Iran

**Keywords:** COVID-19, SARS-CoV-2, diabetes, beta-cell, inflammation, cytokine storm

## Introduction

The COVID-19 pandemic caused by the SARS-CoV-2 coronavirus has become a major threat for our society at multiple levels. SARS-CoV-2 has spread around the world and as per today more than 30 Million people have been infected (World Health Organization). Although it had been suggested at the beginning of the outbreak that a herd immunity with controlled infections should be achieved, we now know of severe complications initiated by SARS-CoV-2, especially critical for metabolic diseases. While COVID-19 mortality is mainly caused by pulmonary failure, a deleterious bidirectional relationship between COVID-19 and diabetes has been observed in several recent studies (1) such that (i) diabetes and the metabolic syndrome are associated with an up to 50% increased risk of fatal and severe COVID-19 for patients with diabetes compared to those who do not have diabetes (2). Furthermore, uncontrolled elevated blood glucose levels are associated with highest COVID-19 mortality rates (3, 4). And (ii) COVID-19 induces severe metabolic complications of pre-existing diabetes such as ketoacidosis and even acute diabetes onset or type 1 diabetes (T1D) (1, 5–9).

### A Human Organoid Platform to Study SARS-CoV-2 Tropism

**Figure 1:**
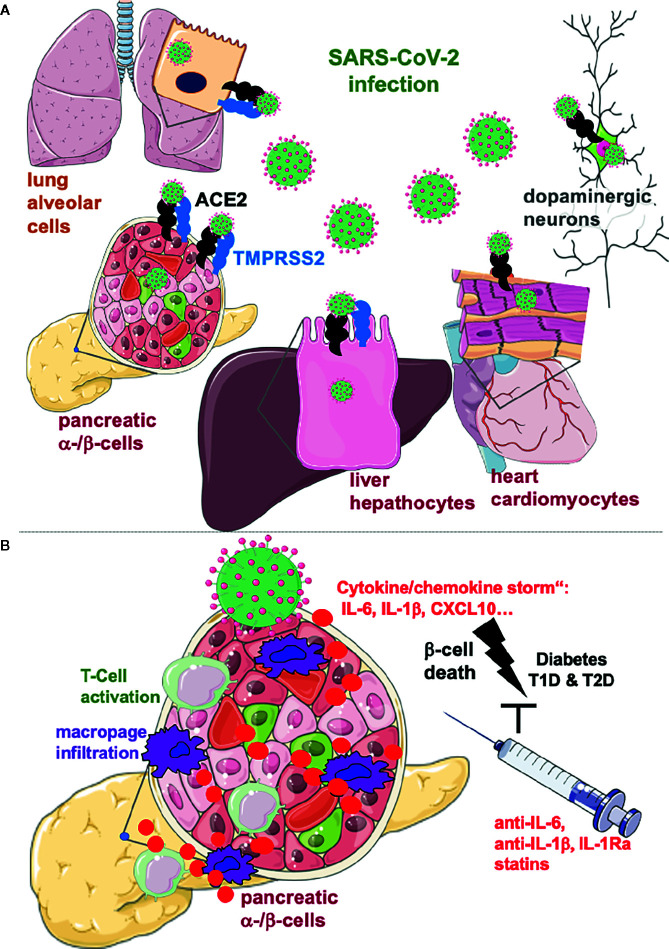
Lung alveolar cells, pancreatic α- and β-cells, hepatocytes, cardiomyocytes, and dopaminergic neurons are specifically permissive to SARS-CoV-2 virus infection. **(A)** SARS-CoV-2 enters cells through the ACE2 receptor and the effector protease TMPRSS2, both expressed on lung alveolar cells and on many other cells in the body, although viral load does not essentially correlate with their expression. Organs affected by the metabolic syndrome are specifically permissive to SARS-CoV-2 infection which is a potential reason for the metabolic deterioration and the more severe disease, seen in COVID-19 patients with obesity and diabetes. **(B)** The “cytokine storm”, i.e. the massive cytokine production by SARS-CoV-2 infected cells may lead to destruction of insulin producing β-cells in the pancreas, which are specifically vulnerable to inflammatory cell death cascades. Consequent activation of cytotoxic CD4^+^ and CD8^+^ T-cells and migration of macrophages may initiate auto-immunity and acute onset of diabetes after COVID-19. Anti-inflammatory therapies, which have been shown to protect β-cells from the vicious cycle of cytokines need to be evaluated for an add-on anti-viral therapy for COVID-19 patients with diabetes or at risk for T2D or T1D. Created using smart servier medical art under https://creativecommons.org/licenses/by/3.0/ .

Yang et al. now provide a unique platform of human cell and organoid models for the SARS-CoV-2 tropism to understand the whole picture of SARS-CoV-2’s effects on various cells and tissues (10). From the tested human pluripotent stem cells (hPSC)–derived cells of all three definitive germ layers, namely, pancreatic α-, β and δ-cells, liver organoids, endothelial cells, cardiomyocytes, macrophages, microglia, and cortical and dopaminergic neurons, they found that pancreatic glucagon-positive α- and insulin-positive β-cells, liver organoids, cardiomyocytes, and dopaminergic neurons are specifically permissive to SARS-CoV-2 virus infection (**Figure 1A**). The potential permissiveness of cells and organoids to SARS-CoV-2 viral entry achieved by a commonly used vesicular stomatitis virus (VSV)-based SARS-CoV-2 pseudo-entry virus with an incorporated SARS-CoV-2 spike protein was confirmed in hPSC-derived primary cells, namely, in human hepatocyte and cholangiocyte organoids and in pancreatic endocrine cells and in isolated primary human islets. Also, an *in vivo* model with hPSC-derived endocrine islet-like cells transplanted under the kidney capsule of humanized mice shows robust SARS-CoV-2 entry and efficiency of virus infection specifically in α- and β-cells of the graft. In primary human islets, SARS-CoV-2 viral infection correlates with the expression of ACE2, the receptor for SARS-CoV-2, expressed in α- and β-cells based on single-cell RNA sequencing (sc-RNA) and immunostaining. The prominent β-cell ACE2 expression was confirmed by three other recent studies, together with its upregulation by inflammation (11) and type 2 diabetes (T2D) (12); a generally slightly higher ACE2 expression was shown in the pancreas in endocrine, acinar and ductal cells, as compared to lung cells (13). In contrast, detailed analyses of RNA-seq data sets revealed very low frequency and expression levels of ACE2 in islets and no differences between non-diabetic and T2D donors (14, 15). In line with the low ACE2 expression in islets, SARS-CoV-2 nucleocapsid protein was primarily expressed in ducts only seen in one out of three investigated autopsy pancreases from COVID-19 patients (14). To resolve the controversy, further detailed histology experiments using the ACE2 antibodies already evaluated in these studies in ACE2 depleted cells are required.

Nevertheless, cellular ACE2 expression itself is not an indicator of SARS-CoV-2 infection in general, as the authors found low or even no viral entry into endothelial cells, macrophages or cortical neurons despite ACE2 expression. Also, the SARS-CoV-2 effector protease, transmembrane serine protease 2 (TMPRSS2), which is currently investigated for antiviral therapy, does not correlate with cellular infection. While highly expressed in the pancreas, it is undetectable in the heart (https://www.proteinatlas.org/), despite viral entry into cardiomyocytes (10). Thus, based on all these recent studies, the role of cellular ACE2 expression in the pathophysiology of COVID-19 remains unclear.

### COVID-19 and Diabetes: An Inflammatory Round Trip

The mechanism for the mortal relationship of COVID-19 and diabetes has been suggested to be associated with the SARS-CoV-2 induced “cytokine storm” through the excessive interferon response to inhibit viral replication. However, if overly, it induces massive tissue damage. Transcriptomic analysis of SARS-CoV-2 virus infected hPSC-derived pancreatic endocrine cells as well as hepatocyte organoids confirmed this hypothesis with the striking upregulation of classical viral sensitive pathways, namely chemokine and cytokine response, apoptotic cell death genes and marked downregulation of key signaling transcripts associated with hormonal functions of endocrine cells, including calcium signaling pathways or key hepatocyte metabolic markers of the Cytochromes P450 superfamily of enzymes in the liver. Notably, cytokine and chemokine profiles linked to the viral response are consistent with those upregulated in lung autopsy samples from COVID-19 patients compared to control donors, including CXCL5, CXCL6, CXCL10, and IL-1β (10). However, all data are based on RNA analyses and it will be important to confirm cytokine protein expression and secretion in future studies.

## Discussion

The concept of virus associated diabetes is not new. Already back in 1926, Franklin Adams observed that severe T1D breaks out “immediately after such an infection”, and this has been later confirmed in many large studies (16). There is a large body of evidence for enteroviral infection initiating the auto-immune response and subsequent β-cell destruction in genetically predisposed individuals, where a viral response is boosted through massive production of pro-inflammatory cytokines. Decorated with a high concentration of pattern recognition receptors, i.e., TLR3/4, RIG-I, MDA-5, and with IL-1R1, and especially vulnerable to inflammatory destruction, the β-cell undergoes apoptosis in response to inflammation, metabolic control derails and diabetes develops. In parallel, the virus initiated interferon response accelerates hyperexpression of surface HLA-I molecules and thus activation of auto-reactive T-cells and auto-immunity against β-cells (16). Also, β-cell failure in T2D has been associated with chronic subclinical inflammation and elevated cytokines and chemokines, especially IL-1β, IL-6, and CXCL10 levels in proximity to the islet are initiator for β-destruction, dysfunction, and metabolic deterioration (**Figure 1B**) (16, 17).

Furthermore, highly elevated serum IL-6, IL-8 and TNF-α in patients with COVID-19 are strong and independent predictors of COVID-19 severity and survival (18), which suggests the „cytokine storm“ indeed as crucial target for therapy. As neither effective antiviral drugs nor approved effective vaccination are currently available, major efforts are needed to treat the deleterious effects of SARS-CoV-2 infection, especially for patients at high risk to develop diabetes; T2D and T1D, obese patients with impaired insulin sensitivity or patients with a family history of auto-immunity. The powerful current state-of-the-art anti-inflammatory corticosteroid therapy (e.g., dexamethasone) needs to be carefully evaluated in patients with diabetes, because of their well-known secondary diabetogenic adverse effects at high doses. Rather, specific and promising anti-inflammatory compounds, such as anti-IL-6, anti-IL-1, and IL-1Ra, which showed robust reduction in markers of inflammation and improvement in glycemia in many clinical trials (17), or lipid lowering anti-inflammatory statins (19) should be considered for the symptomatic therapy for COVID-19 patients with diabetes (**Figure 1B**).

Since investigations have been performed in iPS-derived islet cells or human islets *in vitro*, immediate next steps would be to get access to more autopsy samples from COVID-19 patients, especially from the heart, liver, and pancreas (1) to analyze the virus permissiveness and infection efficiency in these organs and (2) to characterize virus infected cells in patients.

## Author Contributions

AA and KM wrote this article. All authors contributed to the article and approved the submitted version.

## Funding

German Research Foundation (DFG) and JDRF.

## Conflict of Interest

The authors declare that the research was conducted in the absence of any commercial or financial relationships that could be construed as a potential conflict of interest.
